# Certification of NIST SRM 1961: 30 μm Diameter Polystyrene Spheres

**DOI:** 10.6028/jres.096.032

**Published:** 1991

**Authors:** Arie W. Hartman, Theodore D. Doiron, Gary G. Hembree

**Affiliations:** National Institute of Standards and Technology, Gaithersburg, MD 20899

**Keywords:** electron microscopy, microspheres, optical microscopy, particle size, polystyrene, standard reference materials

## Abstract

This report describes the certification of SRM 1961, an NIST Standard Reference Material for particle diameter. It consists of an aqueous suspension of monosize 30 μm diameter polystyrene spheres. The primary technique used optical microscopy; it gave a mean diameter value $\bar{D}=29.62\pm 0.04\;\mathrm{\mu m}$ and a standard deviation of the size distribution *σ_D_* = 0.21 μm. Over 2000 spheres were measured. The supporting technique used electron microscopy, which yielded $\bar{D}=29.68\pm 0.11\;\mathrm{\mu m}$. Ninety spheres were measured.

## 1. Introduction and Summary

This report contains the procedures, measurement results, and error analysis for the certification of SRM 1961, a Standard Reference Material for particle diameter. The SRM consists of a 0.5% aqueous suspension of monosize polystyrene microspheres with a nominal mean diameter of 30 μm.

The calibration was carried out by two independent methods: specialized forms of optical and electron microscopy. The first method is referred to as Center Distance Finding, or CDF; the second method is named Metrology Electron Microscopy, or MEM. The two methods are described in Sec. 2, the measurement results are shown in Sec. 3, and the error analysis is given in Sec. 4.

The results of the calibration are as follows:
mean diameter: 
$\bar{D}=29.62\pm 0.04\;\mathrm{\mu m}$diameter distribution: Gaussian from 3 to 97%Standard deviation *σ_D_* = 0.21 μmnumber of outliers (defined as 
$\left|D-\bar{D}\right|>4\;\sigma_{D}$):1% for oversize1% for undersizeTen samples totaling over 2000 spheres were measured.

## 2. Methods

The two methods used in the calibration of SRM 1961 are described next.

### 2.1 Optical Microscopy (CDF)

**Figure 1:**
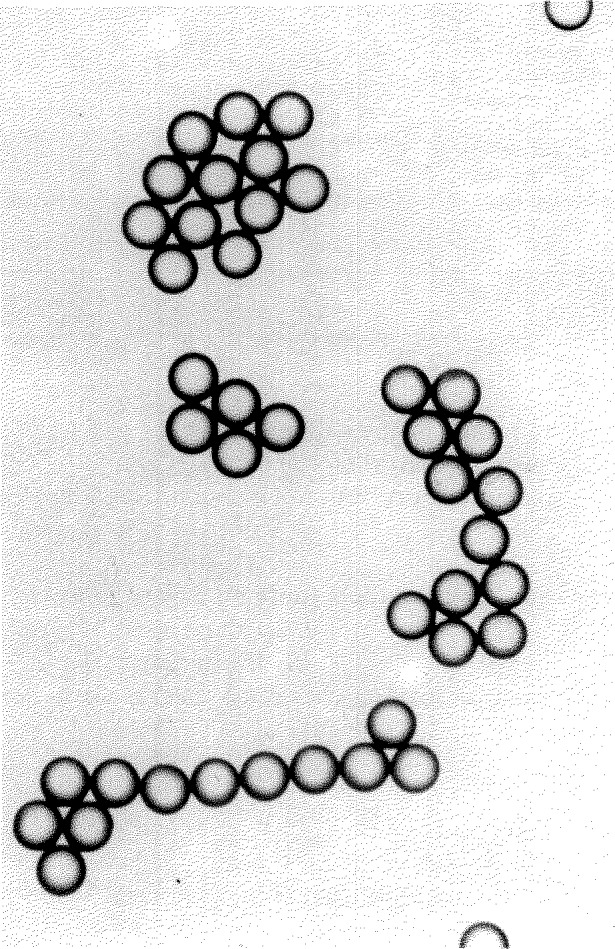
Strands and clusters of 30 μm spheres.

**Figure 2:**
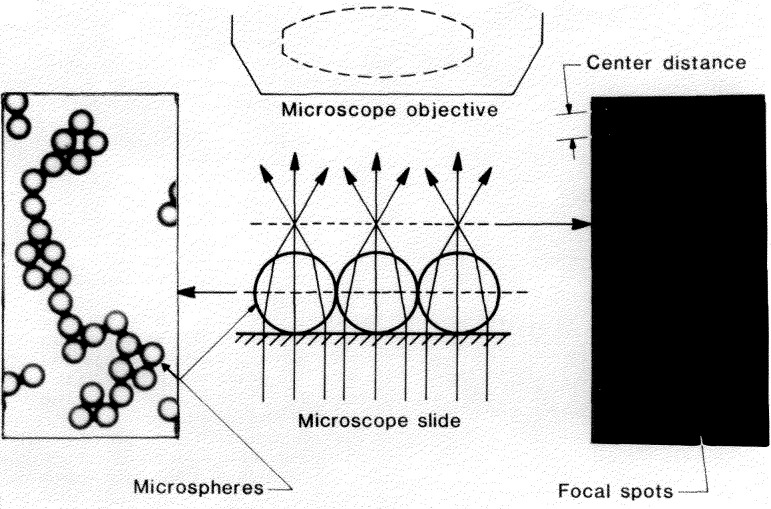
The CDF microsphere sizing scheme.

A drop of microsphere suspension is placed on a microscope slide, allowed to flow out and dry. During drying the drop breaks up into numerous smaller droplets that dry individually. The spheres that these droplets contain are pulled together by surface tension forces, resulting in strands and small clusters of contacting spheres (Fig. 1). The contacting spheres are illuminated by near-parallel light (condenser stopped down), and as shown in Fig. 2 a number of small and circular “focal spots” form in the common back-focal plane. When a photomicrograph is taken of this back-focal plane, each recorded spot marks a sphere center. The distances *C* between adjacent spots represent the sum of two sphere radii. If the sphere diameters *D* are distributed normally (Gaussian), the *C*-values will be distributed normally also. The mean value $\bar{C}$ then equals $\bar{D}$ and the standard deviation *σ_C_* of the *C*-distribution equals $\sigma_{D}/\sqrt{2}$. In this way $\bar{D}$ and *σ_D_* are found. This technique is called Center Distance Finding, or CDF [1].

**Figure 3:**
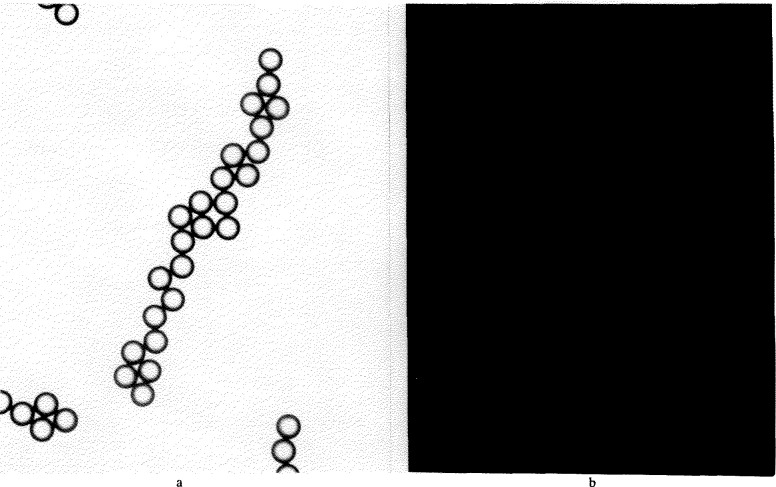
a) A microsphere grouping. b) Its focal spot pattern.

As shown in Fig. 3 the focal spots are small (about 1 μm in the object plane), uniform, and circular, permitting center distances *C* to be measured with high precision: a few hundredths of a μm in the object plane. It thus allows a measurement of the diameter distribution, which would be difficult to do from measurements of the sphere images themselves.

For the measurements, a number of microsphere slides are prepared and photographed. A large number of photographs are measured under computer control (see Appendix A). The film scale (image magnification) is measured, as outlined in Appendix B. The image distortion, which for high-quality optics is a function of off-axis distance only, is measured also (see Appendix B). The computer then applies a radial correction to each measured focal spot position. The corrected center distances *C* are determined, which leads to $\bar{D}$ and *σ_D_*.

### 2.2 Electron Microscopy (MEM)

**Figure 4:**
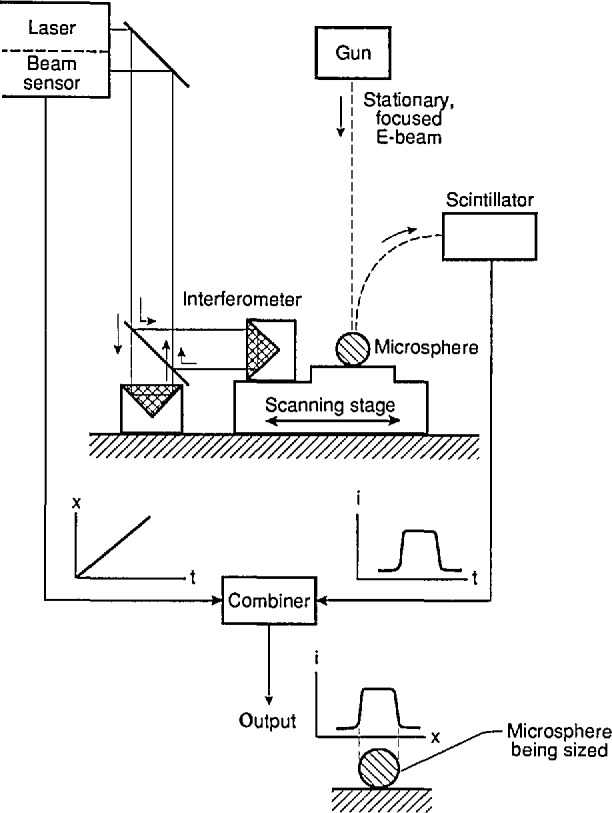
Diagram of the Metrology Electron Microscope (MEM).

With this method, called Metrology Electron Microscopy or MEM, the focused beam of a scanning electron microscope (SEM) is held stationary while a single-axis scanning stage with interferometric position readout moves the specimen such that the electron beam traverses a diameter of a sphere to be measured. An interferometer system measures stage travel versus time during a constant-speed scan, and the secondary electron detection system measures the electron output varying with time, all under computer control. The two data streams are combined, resulting in a value for the edge-to-edge diameter of the sphere [2]. The operation resembles that of an optical measuring microscope, where a set of crosshairs defines a stationary reference point in the field of view and a micrometer screw measures stage travel. See also Refs. [2] and [3], and Fig. 4.

## 3. Measurements

In this section are given details of the specimen preparation, data collection and reduction, and the measurement results. Section 3.1 covers optical microscopy, Sec. 3.2 treats electron microscopy.

### 3.1 Optical Microscopy (CDF)

Four samples were taken from one vial of SRM 1961 microsphere suspension, and one sample from each of three other vials. The vial contents were homogenized by rolling and shaking for two minutes, prior to dispensing a drop of suspension for analysis.

The microscope used was an Olympus Model BH-2^2^ with a 20 × /0.46NA objective, producing images with 200 × magnification on 4 × 5 in Polaroid sheet film.

**Figure 5:**
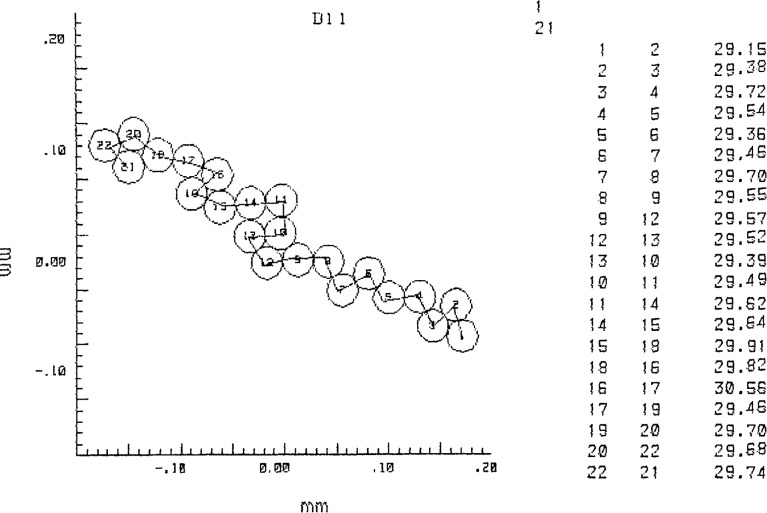
Measurement path for the sphere grouping of Fig. 3.

Focal spot patterns from the contacting microsphere structures were photographed on Polaroid Type 57 positive film. This high-speed material (3000 ASA) has adequate dimensional stability [1] and low granularity, permitting its use for this SRM calibration. Ninety-nine photographs were taken, containing over 2000 focal spots. The center distances between adjacent focal spots were measured by means of a coordinate measuring machine (see Appendix A). The measurement path through each microsphere grouping was selected such that each sphere was measured only once. The groupings of contacting spheres were examined first for overdeterminedness, to indicate where small air gaps between apparently contacting spheres could have formed during the drying process. Such gaps have minimum widths ranging from 0 to typically 1 *σ_D_* [4, 5]. Air gap formation can occur in microsphere groupings, such as hexagonal arrays where six neighboring spheres surround a center sphere while the spheres have slightly different diameters. Such sphere groupings were avoided in the measurement phase. An example of a measured microsphere grouping is shown in Fig. 3, while a selected measurement path is given in Fig. 5.

The measured photographs had a nominal print magnification of 200 ×. The measured focal spots had 0.2–0.3 mm diameters, their 6 mm center spacings were measured with 0.01 mm resolution. The microscope image calibration for magnification and image distortion is detailed in Sec. 4.1.1 and in Appendix B.

**Figure 6:**
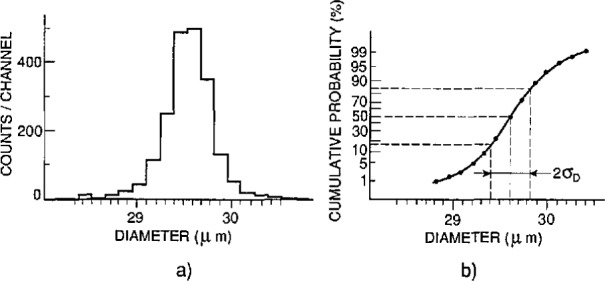
SRM 1961: a) Diameter distribution; b) Cumulative distribution.

Measurement results are given in Table 1 and in Fig. 6. The data were originally plotted with center distances as the horizontal axis. This was then converted into a diameter scale by compressing the horizontal scale by $\sqrt{2}$ to reflect the fact that for normal distributions $\sigma_{D}=\sigma_{C}\;\sqrt{2}$, and by centering the *D*-scale such that the mean diameter $\bar{D}$ coincides with the mean center distance $\bar{C}$. As assumed above, the resulting “diameter distribution” of Fig. 6b already implies that this distribution is considered a normal one. The information extracted from Fig. 6 is: a) the median diameter (which corresponds with the average diameter $\bar{D}$ if the distribution is normal), b) the diameter range over which it actually is normal, and c) the value for the standard deviation *σ_D_* associated with that diameter range.

Sample 1 was covered by four photomicrographs, each containing one large microsphere grouping. It was not possible to select a measurement path for each grouping such that each measured sphere had only two (or three) neighbors. As a result, the structures were likely to show the effect of air gaps present between many visually touching spheres, which increases the measured center distances. In hexagonally ordered microsphere structures (“hexagonal arrays”) the mean value of these gaps is known to be about 0.45 *σ_D_* [4, 5], amounting to 0.10 μm. In large random clusters, such as the four measured, the average gap width could be expected to be comparable to that value. This appears to be the case for sample 1 in Table 1. Excluding that sample lowers the grand mean diameter by 0.01 μm.

### 3.2 Electron Microscopy (MEM)

The contents of an SRM vial 1961 were homogenized by rolling and shaking for 2 min. Then a drop was taken from the vial, diluted in 50 ml of 18 MΩ cm deionized water, and washed three times to reduce the amount of dissolved material remaining (biocide). Each washing cycle involved low-power ultrasonication, settling, and decanting four-fifths of the clear liquid. A small drop of the final suspension was placed on three thin carbon foils supported by 200 mesh copper TEM grids. The grids were then coated with about 30 nm of amorphous carbon to minimize charge-up in the electron beam.

The electron microscope used for the microsphere diameter measurements is a Vacuum Generators VG HB-50A scanning electron microscope. It has in the secondary electron imaging mode an edge resolution of 0.03 μm at 30 keV and a 25 mm working distance. The interferometer is a Hewlett-Packard Model 5526A, utilizing a two-frequency stabilized He-Ne laser and a heterodyne scheme for measuring optical path differences. The two reflectors are mounted in the SEM vacuum on the fixed and moving parts of a piezo-electric one-axis scanning stage. The reflectors are corner cube prisms, to accommodate any misalignment over the relatively long distance (some 80 cm) from the stage inside the SEM column to the interferometer readout system outside. The scanning stage is placed on top of the X-Y stage in the SEM. The X-Y stage is used for searching. A simplified diagram of the setup is shown in Fig. 4.

**Figure 7:**
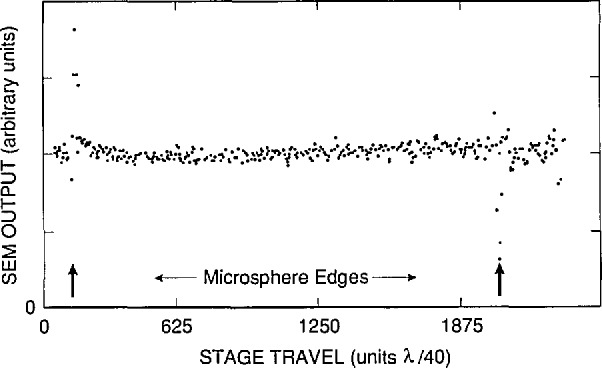
A microsphere scan obtained by MEM.

Thirty microspheres were measured on each of three grids. We selected spheres that were touching one of the copper grid bars as further insurance against beam charge-up. Obvious outliers were excluded from the measurements. After each computer-controlled scan across a microsphere the microscope was reverted to scan mode (SEM) and the next sphere positioned manually for a line scan (spot mode). The scans, of which Fig. 6 shows an example, were about 38 μm long. The secondary electron intensity profile was sampled at 500 equally spaced points. The transition at the sphere edges fell within one data point spacing. The overall shape of this profile was complex, therefore the edge-finding algorithm was simplified by first taking the derivative of this profile and then finding the edges at the most positive and negative values, respectively [6] (see also Fig. 7). Measurement results are given in Table 2.

## 4. Error Analysis

In this section sources of uncertainty (called “errors” for short) are identified and evaluated for the two microsphere size measurement techniques. They are expressed as “3 *σ*” or “maximum” errors as indicated, the individual contributions are summed in quadrature, and the total systematic and random errors are added linearly to form “the uncertainty” of the measurement process (see also Tables 3 and 4).

### 4.1 Errors in Optical Microscopy

The errors in measuring the average diameter can be arranged in three classes: errors associated with finding the image magnification of the measured photographs, errors associated with measuring photographed focal spot spacings (center distances between contacting spheres), and errors associated with the diameter distribution. To find estimates for these errors, five repeat photographs were taken. Averaging of the repeat data was done to reduce the uncertainty of the measured magnification, while comparison between the photographs was used to find scatter in measured focal spot spacings from which uncertainties in the magnification and in a single measurement of center distance can be derived. The three groups of errors are discussed next.

#### 4.1.1 Errors Associated with Image Magnification

The print magnification was found by photographing a calibrated chrome-on-glass stage micrometer (NIST No. 5525). The line center spacings in the prints were measured on a SGIP Universal Measuring Machine, Model MU-214B. The measured and averaged lengths were corrected for image distortion, which had been measured separately (Appendix B). The result was an image magnification value valid over the whole field of view; this value is equal to the on-axis value prior to image-distortion removal. A number of error sources affected the result, as detailed below.

- The object micrometer.
  The distance between the 1.80 and 2.20 mm lines of the calibrated stage micrometer was used. The length of section 0–1.80 mm was 1801.23 μm with a maximum error of 0.07 μm, that of section 0–2.20 mm was 2199.51 ± 0.11 μm, giving for 1.80–2.20 mm a length 398.28 ±0.13 μm. This corresponds to ± 0.033% or 0.010 μm for a 30 μm length in the object plane — a systematic error.
- The SGIP film measuring machine.
  The scale error amounted to approximately 1.2 μm maximum per setting or about 2 μm for a difference between two settings. For the measured film distances (80.491 mm mean value) this amounts to about 0.002% or 0.001 μm in the object plane, a systematic error.
- Film emulsion shifts, image magnification scatter, and film readout errors.
  Polaroid Type 57 positive film exhibits, like most photographic emulsions, local random emulsion shifts. These lateral shifts are caused by non-uniform film processing and drying.
  Magnification scatter is caused by slight changes in film position in the cassette (measured along the optical axis) when exposed film is replaced by a new film sheet.
  Film readout errors reflect the precision with which one can visually pinpoint the center position of the scale division lines of the photographed object micrometer.
  The combined contribution by these three error sources, based on an average of five repeat photographs, was found as follows. The 0.40 mm section of the calibrated object micrometer was photographed repeatedly at 200 ×, giving image lengths (in mm): 80.754, 80.777, 80.719, 80.853, and 80.818. The mean was 80.784, with a 3 *σ* scatter of 0.079 mm or 0.10%. This total scatter contributes a 0.030 (μm systematic error to a 30 μm center distance measurement. After correction for image distortion (see part d) the mean becomes 80.471 mm, giving an on-axis image magnification *M*_o_ = 80.471 mm/398.28 μm = 202.0 ×.
- Image distortion.
  The microscope exhibits radial image distortion: each off-axis image point is shifted radially by a small amount from its true position. In our case each end point of the 80 mm measured length was shifted outwards by 0.15 mm typically, with an estimated maximum uncertainty of 0.015 mm (Appendix B). This amounts to a combined 0.030 mm uncertainty in the measured length (the two error contributions are correlated), or 0.04%, corresponding to 0.012 μm in the object plane.

The error contributions a) through d) combine to a total systematic error of 0.034 μm (see Table 3).

#### 4.1.2 Errors Associated with the Determination of Microsphere Center Distances

The accuracy of center distance measurements is affected by various uncertainties: (a) those associated with pinpointing the positions of focal spot centers in the film, (b) with the correction of measured focal spot positions due to image distortion, (c) with the fluctuations in print magnification when new film is inserted in the cassette, (d) with possible distortion of the spheres at the contact areas, and (e) with the possibility that the individual spheres might be slightly deformed (showing a non-circular cross section when measured perpendicular to the line of sight).

- The combined effect of film readout (pinpointing sphere centers) and emulsion shifts was found by taking five repeat exposures of a hexagonal array of the 30 μm spheres and measuring each time the same 17 distances between adjacent sphere centers in a selected microsphere row. This was done under computer control as described in Appendix A. All sets of five readings each were scaled down to the same average value (nominally 6.0 mm as a result of 200 × magnification of the 30 (μm spheres). The 85 values were then pooled, resulting in a total scatter of 43 μm which amounts to 0.22 (μm in a 30 μm object distance. This is a random error. As can be seen, this procedure reduced the effects of magnification scatter and avoided the effects of off-axis magnification changes due to image distortion, and of unequal-size spheres.
  When pinpointing the center positions of the focal spot recordings in the film, the utilized coordinate measuring machine with TV-microscope probe exhibited a reproducibility of better than 0.5 μm at 1 *σ* (see Appendix A). It translates to a maximum error of 2 μm in film distances between two focal spots, or 0.01 μm in distances between microsphere centers. This random error does not increase the 0.22 μm random error calculated above.
- Magnification scatter, occurring when replacing sheet film in the cassette, was measured as 0.27% at 3 *σ* for the central area of a single exposure (see Appendix B). This value is considerably larger than can be expected from the data in paragraph c) of Sec. 4.1.1. One reason for this is that paragraph c) relates to measurements near the edges of the film sheet (the image of the object micrometer segment spans the field of view), where it is clamped by the cassette mechanism and consequently flexes much less. The corresponding maximum error for a 30 μm center distance measurement is 0.08 μm, an essentially random error. It has been applied to all areas in the film, as a worst case.
- The effect of image distortion in our case (see Appendix B) is maximum for a sphere pair at the edge of the measured field of view. At 40 mm off-axis distance the maximum error in the measured image distortion is about ± 15 μm; at 34 mm it is ± 10 μm. Assuming that these errors are uncorrelated (a worst case), the resultant maximum error for 6 mm center distances oriented radially near the edge of the 80 mm field of view will then be ± 18 μm or 0.09 μm in the object plane, a random error. For center distances closer to the optical axis this error will be considerably less, and for those on the axis the error will be zero.
- One can adopt the model that two polystyrene spheres approaching each other during the drying process will finally be in intimate contact over a circular area, the extent of which is controlled by a balance between van der Waals attraction and elastic deformation. This model has been analyzed by Derjaguin et al.; they have derived an expression for the resultant sphere flattening [7]. For the present case the two-sided flattening would amount to a shortening Δ*C* of the measured center distance C given by
  $$\Delta C=\frac{1}{8}{\left[\frac{6{\left(1-\eta^{2}\right)}^{2}DA^{2}}{\epsilon^{4}E^{2}}\right]}^{1/3},\;\text{in}\;\text{which}$$
  η = Poisson constant, 0.3 for polystyrene
  *D* = sphere diameter, 3 × 10^−3^ cm
  *A* = Hamaker constant, 1 × 10^−12^ erg for polystyrene
  *E* = Young’s Modulus, 3 × 10^10^ dyne/cm^2^ for polystyrene
  *ϵ* = distance of closest approach, 3 × 10^−8^ cm
  This gives Δ*C* = 3.4 nm = 0.003 μm, lowering the measured diameter. If the selected values for *A* and *E* are each uncertain by a conceivable factor 2, then Δ*C* could change by a factor whose maximum value is $\sqrt[{3}]{16}=2.5$. The Δ*C* estimate then ranges from 0.001 to 0.009 μm.
  Although this model for sphere flattening on contact is not the only one available [8], experimental data (comparison with other calibration techniques for various monosize microsphere SRMs) support the Derjaguin model. Therefore the measured diameter values in Table 1 include a correction by a somewhat arbitrary increase of 0.01 μm, and a random error 0.009 μm is entered into the error analysis.
- If a microsphere is elongated perpendicular to the line of sight, its focal spot will be elongated by the same amount [4]. The photographed focal spots are almost all very uniform and circular, with a diameter of 0.20 mm in the film plane corresponding to 1.0 μm in the object plane. A non-circularity of 0.03 mm is visually detectable, and any residual non-sphericity will then not exceed 0.15 μm, a random error. The random contributions combine to a maximum random error of 0.29 μm.

#### 4.1.3 Errors Associated With the Microsphere Diameter Distribution

Figure 7 shows that the diameter distribution is not quite normal. Of the measured population, 1 to 99% covers the size range 28.8 to 30.4 μm. The maximum error contribution to a single center distance measurement can be set at ± 0.30 μm, a random error.

#### 4.1.4 Combining the Various Error Contributions for the $\bar{D}$ Measurement

From Table 3 the total random error amounts to $0.41/\sqrt{2000}=0.009$ the total systematic error is 0.035 μm, therefore the total error in $\bar{D}$ is 0.039 μm. The reported value for $\bar{D}$ becomes 29.62 ±0.04 μm.

#### 4.1.5 Finding the Standard Deviation of the Size Distribution

Figure 5 shows that the diameter distribution is normal from 3 to 97% (1900 spheres), and the calculated value of *σ_D_* for this population is 0.23 μm.

- The statistical uncertainty in *σ_D_* based on 900 measurements is ± 10% at 3 *σ*, or 0.023 μm.
- Subtracting in quadrature the 1 *σ* random uncertainty in a single measurement of center distance (equal to 0.26/3 or 0.09 μm; see Table 3) lowers *σ_D_* to 0.21 μm, with an uncertainty of about 0.03 μm.

The reported value for *σ_D_* is therefore *σ_D_* = 0.21± 0.03 μm.

### 4.2 Errors in Electron Microscopy

#### 4.2.1 Errors Associated With Microsphere Sensing

- Imperfect scans and E-beam exposure.
  Each selected microsphere was positioned under the stationary electron beam such that a subsequent computer-controlled scan would result in the E-beam traversing the microsphere across its center as close as possible, while being measured edge-to-edge. Each scan was repeated a total of three times. In order to minimize errors from off-center scans and from a possible slight shrinking of the polystyrene microspheres from E-beam exposure, only the largest of the three edge-to-edge distances for each measured sphere was used in the data analysis.
  The combined effect of these error sources is estimated as 0.1%, or 0.03 μm for a single diameter measurement. Because it is not known which part of this error is random or systematic, the whole error is considered a systematic one.
- SEM spatial resolution and E-beam wander.
  The edge resolution is 0.03 μm, giving for a diameter measurement a random uncertainty of 0.042 μm. The effect from E-beam wander is considered negligible.

#### 4.2.2 Errors Associated With Stage Travel Measurement

- Stage travel sampling.
  The stage travel was 37.9 μm for each scan; it was sampled at 500 equidistant points, giving a digitizing error of ±0.076 μm.
- Interferometer-output digitizing.
  The HP interferometer readout system had a least count of λ/40, giving a digitizing error of ±0.016 μn.

#### 4.2.3 Errors Associated With the Microsphere Diameter Distribution

The diameter distribution has *σ_D_* = 0.21 μm. For 90 measurements the 3 *σ* error in the mean diameter is $0.63/\sqrt{90}=0.066\;\mathrm{\mu m}$.

#### 4.2.4 Combining the Various Error Contributions to $\bar{D}$

Referring to Table 4, the 0.042 and 0.63 μm random errors combine to 0.63 μm. The digitizing errors add linearly to this, giving 0.72 μm. With the systematic error of 0.03 μm the total error in $\bar{D}$ becomes $0.03+0.72/\sqrt{90}=0.11\;\mathrm{\mu m}$, giving a reported value for MEM: mean diameter $\bar{D}=29.68\pm 0.11\;\mathrm{\mu m}$.

## 5. Diameter Calibration Final Results

The results of the certification are as follows:

Mean diameter of the SRM 1961 microspheres:

$\bar{D}=29.62\pm 0.04\;\mathrm{\mu m}$(optical microscopy)*σ_D_* =0.23 ± 0.03 μm(central peak)Supporting value:
$\bar{D}=29.68\pm 0.11\;\mathrm{\mu m}$(electron microscopy)The quoted uncertainties are maximum values.

## 6. Sample Uniformity

From Table 1 an impression of sample uniformity can be obtained: the within-vial variation in the measured average diameter is ±0.1%, and the between-vial variation amounts to a slightly larger amount (±0.13%). Sample 1 was a special case: instead of measuring string-like groupings, where each microsphere can freely touch its neighbors, four large conglomerates were measured (one per photograph). Such structures are overdetermined in the sense that after drying small air gaps can remain between apparently touching spheres. As expected, the average center distance between neighboring spheres was significantly larger for this sample than for all others. Because the average gap width is related to the sphere diameter distribution, a found increase in measured average center distance by about 1/4 *σ_D_* does not seem unreasonable. Taking this into account, only upper limits for the SRM non-uniformity are quoted: ±0.1% for within-vial and between-vial sampling.

## 7. Outliers

As with the sample uniformity, only upper limits could be set to the percent oversize and undersize of the measured 2000 spheres. When outliers are defined as spheres with sizes more than 4 *σ_D_* different from the mean diameters, there are nearly 1% oversize and nearly 1% undersize (Fig. 5b). Spheres that were outsize by some 10% or more could be found by visual inspection of the photomicrographs. In this way two oversize and two undersize spheres were found.

## Figures and Tables

**Figure 8:**
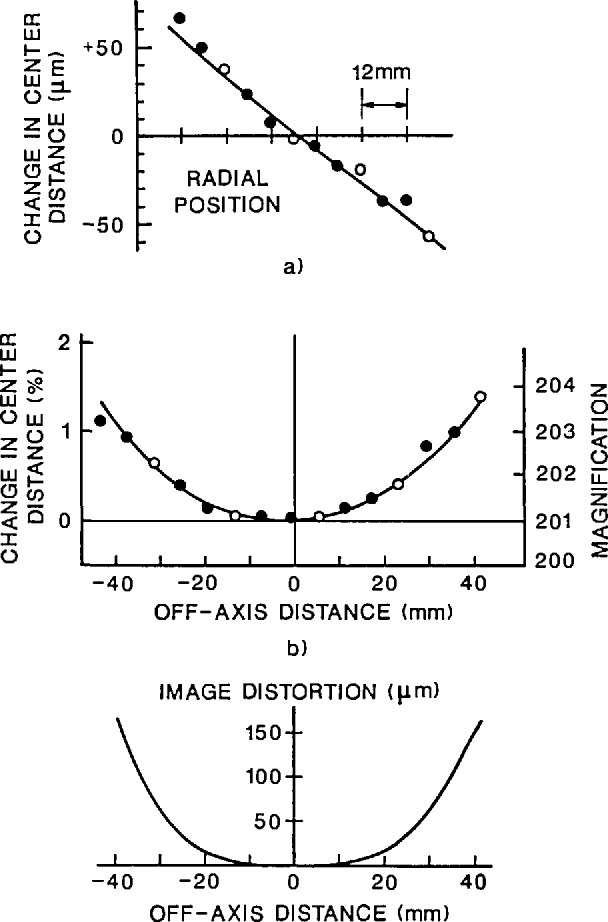
Finding microscope image distribution and magnification.

**Figure 9:**
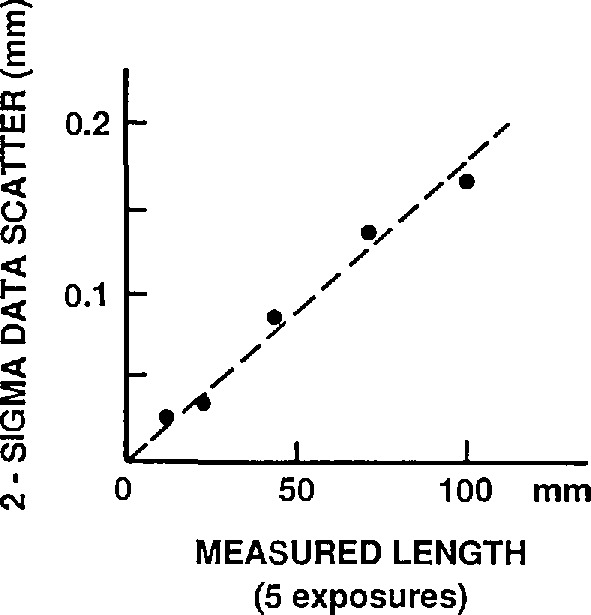
Scatter in image magnification.

**Table 1:** Measurement results with optical microscopy^a^

Vial#	Sample *#*	Sphere diameter (μm)		# of measurements	# of photographs
		average	median		
1	1	29.72	29.72	218	4
1	2	29.66	29.66	192	6
1	3	29.63	29.64	345	28
1	4	29.63	29.64	276	25
1	1 to 4	29.65	29.66	1031	63
2	5	29.59	29.59	608	18
3	6	29.60	29.59	255	12
4	7	29.58	29.57	151	6
all	all	29.62	29.62	2045	99

a Diameter distribution is approximately normal from 3 to 97%. Standard deviation over this interval: 0.21 μm.

**Table 2:** Measurement results with electron microscopy

Area #	Average diameter(μm) $\bar{D}$	Standard deviation(μm) *σ_D_*	# of measurements
1	29.66	0.19	30
2	29.70	0.38	30
3	29.69	0.30	30

average	29.68		

**Table 3:** Error budget for a single 30 μm center distance measurement, using CDF^a^

Category	Error source	Error contribution (μm)	
		Systematic	Random
On-axis magnification	Stage micrometer calibration	0.010	
	Film measuring machine calibration	0.001	
	Film readout, emulsion shifts, and magnification scatter (5 exposures)	0.030	
	Image distortion uncertainty	0.012	

		0.034	
Center distance measurement	Film readout and emulsion shifts		0.22
	Magnification scatter		0.08
	Image distortion-worst case		0.09
	Sphere flattening on contact		0.009
	Non-sphericity		0.15

	**Subtotals**	0.034	0.29
Finite sample size (*N* = 2000)	Diameter distribution width		0.30

	**Totals**	0.034	0.42

a Uncertainty in $\bar{D}:\;0.034+0.42/\sqrt{2000}=0.04\;\mathrm{\mu m}$. Measured $\bar{D}$ (after corrections, see Sec. 4.1.2): 29.62 μm.

**Table 4:** Error budget for a single 30 μm sphere diameter measurement, using MEM^a^

Category	Error source	Error contribution (μm)	
		Systematic	Random
Microsphere sensing	Imperfect scan and E-beam exposure	0.03	
	SEM spatial resolution and E-beam wander		0.042
Length measurements	Stage travel sampling		0.076
	Interferometer output digitizing		0.016
Finite sample size (*N* = 90)	Diameter distribution width (*σ_D_* = 0.21 μm)		0.63

	**Totals**	0.03	0.72

a Uncertainty in $\bar{D}:\;0.03+0.72/\sqrt{90}=0.11\;\mathrm{\mu m}$. Measured $\bar{D}:\;29.68\;\mathrm{\mu m}$.

## Footnotes

2 Certain commercial equipment, instruments, or materials are identified in this paper to specify adequately the experimental procedure. Such identification does not imply recommendation or endorsement by the National Institute of Standards and Technology, not does it imply that the materials or equipment identified are necessarily the best available for the purpose.

## 8. Appendix A. Measuring Microsphere Center Distances from Photomicrographs

The relative positions of the photographed microsphere focal spot positions are read out with a coordinate measuring machine (CMM). Its probe is a microscope containing a high-resolution image sensor (vidicon) and circuitry to pinpoint the center of each focal spot, which is interfaced with the CMM control computer to bring the focal spot center to the boresight axis (center of the microscope’s field of view). The CMM’s *X-Y* coordinates are then stored.

Next, for each photograph being measured a decision is made as to what measurement path will be selected from one focal spot to the next, in order to make sure that each sphere is measured only once and that each sphere in the grouping could have been free to assume its contacting position with its neighbors during drying (no mechanically overdetermined sphere arrangement). The path selection is done by an operator using an interactive graphics routine. The CRT display shows all focal spots in their relative positions with circles drawn around each one, to simulate the actual microsphere scene rather than the focal spot representation of it. It also shows the keyed-in measurement path (see Fig. 5). The result is a string of *X-Y* coordinates in which each increment represents a center distance (CD) between two adjacent (contacting) spheres.

Before the CDs are computed each focal spot position is first corrected for image distortion of the microscope (this distortion can be determined as in Appendix B). Because the distortion is a radial function these position corrections take the form of small radial shifts. Their magnitudes depend on the initial off-axis distances, and are found by means of a stored function.

An impression of the repeatability and accuracy of the film measuring system can be obtained from the following information:

1. The CMM is normally used for the calibration of precision grid plates. It has been extensively studied, and its positional accuracy is better than 0.1 (μm over the area of the pictures measured [9].
2. Each focal spot was centered to the same point of the camera field, and all the spots were about the same size. Therefore any geometrical errors in the camera scan can have only secondary effects on the measurement.
3. A photograph selected at random was run a number of times without being moved. The center distances (6 mm nominally, the print magnification being 200 ×) showed a repeatability with standard deviation less than 0.5 μm.
4. One other photograph was measured repeatedly while the microscope focus was varied in both directions. Since the focal spot brightness was not completely isotropic, the focus settings changed the apparent size and shape of the focal spots by small amounts. The resultant changes in focal spot positions were very small and uncorrelated, with an estimated standard deviation of less than 0.5 μm.
5. A few photographs were reexamined after all had been measured in order to check for any process changes during the measurements. No changes larger than the measured repeatability were found.

The CMM used was a five-axis Moore M5Z from Moore Special Tool Company. The TV camera system consisted of a Dage-MTI Inc. high-resolution vidicon camera Model 65, with a Bausch & Lomb lens type Mono Zoom 7 and a vision system from Videometrix VPU, Model 101110-501-14. The scene illumination was a diffuse one, using a fiberoptic ring illuminator from Titan Tool Company.

## 9. Appendix B. Measuring the Print Magnification (Film Scale) and Image Distortion

To set the scale of the photographs a section of a calibrated object micrometer is photographed and measured. To maximize resolution the photographed length is made to span the whole field of view. The measured length is then corrected for the effects of image distortion which causes off-axis image points to be radially shifted from their intended positions. The corrected length now shows the image magnification in the absence of image distortion. In other words: one has found the on-axis image magnification value.

For the determination of image distortion the microscope magnification itself is not needed. In the following is shown how these properties were measured.

- Image distortion.
  Following a scheme outlined in Ref. [1] this goes as follows. A row of microspheres is placed such that it crosses the center of the field of view. Its row of focal spots is photographed. Then the row is shifted in-line by three sphere diameter (3 *D*) and its focal spots are recorded again. All distance between adjacent sphere centers are measured in both photographs; it will be seen that some center distances will have decreased while others increased. These changes are so small that a 3 *D* line section rather than *D* was chosen in order to make the center distance changes stand out from measurement noise (see Fig. 8a).
  Assuming a 3 *D* shift from left to right, and starting with the far left (first) sphere pair, one finds the accumulated length changes when the center distance length *D* is shifted in-line by 3 *D* at the various points in the field of view. A second data set is found by repeating the length change calculations starting at the next left sphere pair. A third data set is obtained from the third left sphere pair. The three data sets belong to a common curve, and a best fit of all three sets is obtained as shown in Fig. 8b.
  Figure 8b therefore shows by what percentage a center distance *D* will vary as it is shifted all across the field of view (this is equal to the percent change in magnification for radial objects as a function of off-axis distance). A graphic integration then yields the radial shifts of off-axis image points as a function of their off-axis distance, that is, the image distortion as shown in Fig. 8c.
- Film scale.
  With the image distortion known, the film scale or on-axis magnification *M*_0_ is found next using steps already described. If *M*_0_ is measured a number of times the results will show data scatter, typically 0.3% at 3 σ. This is caused by small changes in axial position when fresh sheet film is inserted in the cassette. The corresponding scale changes can be reduced by averaging over a number of repeat exposures, for instance five, of the object micrometer.
  The effect of inserting fresh sheet film in the cassette can be measured by placing a row of microspheres such that it crosses the center of the microscope’s field of view, taking five repeat photomicrographs, and measuring a number of sphere center distances in all photographs. The scatter found in these lengths contains the combined effects of film readout, local emulsion shifts and changes in film scale. As shown in Fig. 9 these data approximate a straight line through the origin; the slope of that line represents the scatter in film scale or magnification (0.09% at 1 σ in our case), for areas near the center of the photographs. For this experiment a 10 μm microsphere array was centered in the field of view, and the various lengths in Fig. 9 were realized by summing the lengths of a number of adjacent microsphere rows. The found magnification scatter was indicative of film flexure at the central area of the film frame.
